# The HIF1α-PDGFD-PDGFRα axis controls glioblastoma growth at normoxia/mild-hypoxia and confers sensitivity to targeted therapy by echinomycin

**DOI:** 10.1186/s13046-021-02082-7

**Published:** 2021-09-01

**Authors:** Gong Peng, Yin Wang, Pengfei Ge, Christopher Bailey, Peng Zhang, Di Zhang, Zhaoli Meng, Chong Qi, Qian Chen, Jingtao Chen, Junqi Niu, Pan Zheng, Yang Liu, Yan Liu

**Affiliations:** 1 Institute of Translational Medicine, the First Hospital of Jilin University, Changchun, Jilin China; 2 Division of Immunotherapy, Department of Surgery and Comprehensive Cancer Center, Institute of Human Virology, University of Maryland School of Medicine, Baltimore, USA; 3 Department of Neurosurgery, Neuroscience Research Center, The First Hospital of Jilin University, Changchun, Jilin China; 4 Beijing Key Laboratory for Genetics of Birth Defects, Beijing Pediatric Research Institute, Beijing Children’s Hospital, Capital Medical University, National Cancer for Children’s Health, Beijing, China; 5 Department of Neurosurgery, Beijing Children’s Hospital, Capital Medical University, National Cancer for Children’s Health, Beijing, China; 6 OncoC4, Inc., Rockville, MD USA

**Keywords:** HIF1α, PDGFRα, PDGF-D, Glioblastoma, Echinomycin

## Abstract

**Background:**

Glioblastoma multiforme (GBM), a lethal brain tumor, remains the most daunting challenge in cancer therapy. Overexpression and constitutive activation of PDGFs and PDGFRα are observed in most GBM; however, available inhibitors targeting isolated signaling pathways are minimally effective. Therefore, better understanding of crucial mechanisms underlying GBM is needed for developing more effective targeted therapies.

**Methods:**

Target genes controlled by HIF1α in GBM were identified by analysis of TCGA database and by RNA-sequencing of GBM cells with HIF1α knockout by sgRNA-Cas9 method. Functional roles of HIF1α, PDGFs and PDGFRs were elucidated by loss- or gain-of-function assays or chemical inhibitors, and compared in response to oxygen tension. Pharmacological efficacy and gene expression in mice with intracranial xenografts of primary GBM were analyzed by bioluminescence imaging and immunofluorescence.

**Results:**

HIF1α binds the *PDGFD* proximal promoter and *PDGFRA* intron enhancers in GBM cells under normoxia or mild-hypoxia to induce their expression and maintain constitutive activation of AKT signaling, which in turn increases HIF1α protein level and activity. Paradoxically, severe hypoxia abrogates PDGFRα expression despite enhancing HIF1α accumulation and corresponding PDGF-D expression. Knockout of *HIF1A*, *PDGFD* or *PDGFRA* in U251 cells inhibits cell growth and invasion in vitro and eradicates tumor growth in vivo. *HIF1A* knockdown in primary GBM extends survival of xenograft mice, whereas *PDGFD* overexpression in GL261 shortens survival. HIF1α inhibitor Echinomycin induces GBM cell apoptosis and effectively inhibits growth of GBM in vivo by simultaneously targeting HIF1α-PDGFD/PDGFRα-AKT feedforward pathway.

**Conclusions:**

HIF1α orchestrates expression of PDGF-D and PDGFRα for constitutive activation of AKT pathway and is crucial for GBM malignancy. Therefore, therapies targeting HIF1α should provide an effective treatment for GBM.

**Supplementary Information:**

The online version contains supplementary material available at 10.1186/s13046-021-02082-7.

## Background

Glioblastoma multiforme (GBM) is the most common primary aggressive brain tumor, causing death within two years after diagnosis despite current therapies [1]. Malignant GBM is referred to as grade IV astrocytic glioma, based on WHO classification of four histology grades comprised of pilocytic, diffuse, anaplastic astrocytomas and glioblastoma, and incorporated with molecular genetic features for diagnosis [2–4]. Based on gene mutations and molecular profiling, GBM is divided into four subtypes including proneural, neural, classical and mesenchymal GBMs [4, 5]. Most proneural GBM, the most resistant subtype, have mutations in *TP53* in conjunction with overexpression of PDGFRα [5]. Integrative genetic analysis has demonstrated that 88% of GBM is caused by constitutive activation of the receptor tyrosine kinase (RTK)/RAS/PI3K signaling pathways and defective RB and/or ARF-p53 signaling pathways [6–8]. Genes encoding epithelial growth factor receptor (EGFR) and PDGFRα were altered by amplification, rearrangements and mutations, resulting in increased receptor tyrosine phosphorylation in GBM [9]. In adult GBM, *EGFR* amplification is the most frequent alteration (45–57% of cases), while *PDGFRA* amplification is the second most frequent (10 to 20% cases). The incidence of *PDGFRA* amplification increases to 23% in pediatric GBM [10] and 30% in high-grade pediatric gliomas [9, 11, 12]. Other genetic lesions, including *PDGFRA* activating mutations and gene rearrangements, as well as *EGFR* amplification, often occur concurrently in tumors with *PDGFRA* amplification [11–15]. However, overexpression of *PDGFRA* was detected in majority of proneural subtype GBM, which was substantially more frequent than *PDGFRA* genetic alterations [5]. Meanwhile, genetic alterations of components of the PDGFRα-PI3K-AKT signaling pathway occur in up to 70% of GBM [16]. Moreover, co-overexpression and co-activation of PDGFRα with EGFR often occur in GBM tumors without amplification of either gene [17–19] but with a typical feature of high angiogenesis such as the most common EGFRvIII mutant-overexpressing GBM [20, 21].

HIF1α is stabilized under hypoxic conditions and responsible for directing tumor angiogenesis. Hypoxia inactivates the prolyl-hydroxylases in cytosol and the arginine hydroxylase factor inhibiting HIFα in nucleus, leading to the prevention of recognition and degradation of HIFα by the E3 ligase Von Hippel-Lindau, and to the inhibition of HIFα transcriptional activity, respectively [22–24]. In contrast, we demonstrated an essential role of HIF1α under normoxia in leukemia/lymphoma stem cells, which is efficiently targeted by echinomycin, an inhibitor of HIF1α transcriptional activity [25, 26]. Glioblastoma typically features three-layers including a necrotic core, intermediate/hypoxic layer, and a well-oxygenated and -vascularized, highly-proliferative outer layer comprising the invasive tumor frontier [27, 28]. HIF2α is required for the growth of glioma stem cells at hypoxia [29]. Although HIF1α is highly expressed in both glioma stem and bulk tumor cells [29], its role has not been thoroughly evaluated.

The family of PDGFs and PDGFRs is comprised of PDGF-A, B, C and D, and PDGFRα and β. Overexpression of PDGF-A, B, and C has demonstrated that PDGF-PDGFR signaling plays an important role in both normal development and tumorigenesis of the central nervous system (CNS) [30, 31]. PDGFRα is expressed predominantly in glial progenitors and has a reduced expression in mature astrocytes [32]. In mice, PDGFRα overexpression, together with the loss of ARF, was reported to induce GBM via PDGFRα-PI3K-AKT activation [33]. PDGFRβ is mostly restricted in the glioma-associated stroma, but can be induced in glioma cells by microglia to enhance the migration of glioma cells [34, 35]. Its ligands, PDGF-B and PDGF-D, were both shown to be more potent mitogens for the growth and transformation of fibroblast cells than PDGF-A and PDGF-C [36, 37]. Overexpression of PDGF-B in glial progenitors of transgenic mice induced gliomas in a longer latency, and high-grade gliomas at a shorter latency once combined with Arf or Trp53 deficiency [38, 39]. However, the expression, function, and regulation of PDGF-D in CNS cells remains less known.

Here, we demonstrate that HIF1α plays a critical role in favoring the growth of GBM cells via directly inducing the expression of both PDGF-D and PDGFRα for constitutive AKT activation, which primarily occurs at normoxia or mild-hypoxia. The induced PDGF-D is essential for GBM growth in vivo via an autocrine and/or paracrine manner, to increase tumor invasion and angiogenesis in mouse models of GBM.

## Materials and methods

### Mice, cells and reagents

#### Mice

*Nod.Scid.Il2rg*^*0*^ (NSG) mice were purchased from the Jackson lab. Mice at 6–8 weeks were used for the intracranial implantation of glioblastoma cells and for the treatment. All experiments were performed using mycoplasma-free cells.

#### Cell lines and primary GBM cells

The human GBM cell lines U251 and U87MG and mouse GBM line GL261 were obtained from the Cell Bank of Chinese Academy of Sciences (Shanghai, China). These GBM cell lines were cultured in high-glucose DMEM supplemented with 10% FBS, 2 mM glutamine and penicillin/streptomycin (100 U/mL, each), and maintained at 37 °C, 5% CO2 incubator. Unless stated otherwise in the figures or figure legends, GBM cells were cultured at normoxia (21% oxygen).

Primary LGG and GBM samples were obtained from surgical-resections of adult gliomas at the First Hospital of Jilin University between Jan 2013 and Jun 2014 (Supplemental Table 1). The Ethics Committee of the Jilin University approved and patient consent was provided for this study. Two primary GBM tissues obtained from surgical resections were immediately placed in RPMI 1640 medium. The tissues were chopped into a fine paste with scalpels and digested in 0.25% trypsin/EDTA solution containing 100 U/mL DNase I (Sigma-Aldrich) for 30 min at 37 °C with 125 rpm shaking, and terminated by adding 10% FBS DMEM medium. Digested cells were pelleted by centrifugation, gently suspended with 1 mL of 1x lysing buffer solution (BD Bioscience) for a total of 2 min, neutralized with 10 mL DMEM medium, passed through a 70 μm filter, and pelleted by centrifugation. The isolated cells were cultured in NeuroCult basal medium containing 1x differentiation supplement (Stem Cell Technology), 40 ng/mL EGF (Invitrogen), 20 ng/mL b-FGF (Invitrogen), 1 mM glutamine, and 1x antibiotics. The primary cells at less than three passages were used for in vitro assays and the freshly separated tumor cells from mice were used for in vivo assays and tumor implantation.

#### Reagents

Rabbit HIF1α antibody (GTX127309, GeneTex), Phospho-Y754-PDGFRα (Ab5460, Abcam), Phospho-AKT(S473) and AKT, Phospho-ERK(T202/Y204) and ERK, Phospho-EGFR (Y1068) and EGFR, and PDGFRβ (28E1) all from Cell Signaling Technology; rabbit PDGFRα (C20, sc338), mouse monoclonal PDGFRα (C9, sc-398,206) and p53 (DO-1, sc-126), and rabbit GAPDH (FL-335, sc-25,778) all from Santa Cruz, were purchased. Goat PDGF-D (AF-1159) antibody for human, PDGF-DD ELISA kit (DDD00) and all PDGF growth factors were purchased from R&D Systems. Mouse CD31 antibody (JC/70A, MA5–13188) and rabbit PDGF-D (40–2100) from Invitrogen, rabbit PDGF-B (28E1, AF0204) from affinity Biotech, beta-actin (AC-74) antibody and puromycin from Sigma, and the PDGFR inhibitor AG1296 and the EGFR inhibitor AG1478 from Cayman Chemical, were purchased.

#### Plasmids

The full-length coding cDNAs of *HIF1A* from human bone marrow cells and of *PDGFRA* and *PDGFD*, both from U251 cells, were cloned into a pcDNA vector and identified by DNA sequencing. The triple *HIF1A* mutant form (P402A/P564A/N802A, named as HIF1α-PPN) was made by site-directed mutagenesis using *HIF1A* as template and a kit from Clontech. The PDGFD-dCUB construct was made by deletion of the CUB domain of PDGFD using Platinum SuperFiII DNA polymerase (Invitrogen) and ligated with T4 ligase (Promega).

### Cas9/CRISPR gene knockout in glioblastoma cells

Design of small Cas9-guided RNA (sgRNA) sequence was based on the website tool of Zhang’s lab (http://crispr.mit.edu/). Knockout of *HIF1A* or *PDGFD* or *PDGFRA* genes was performed in U251 or U87MG cells by transfecting the sgRNA-expressing plasmid of *HIF1A* or *PDGFD* or *PDGFRA* or control scrambled sg (Sr-sg). The plasmid is constructed in a Lenti-Crisp-V2 vector (addgene, Cambridge, MA) and expresses both Cas9 protein and sgRNA of the DNA sequence of *HIF1A* (5-ccatcagctatttgcgtgtg-3) or *PDGFD* (5-ctttgcgcaacgccaacctc-3) or *PDGFRA* (5-cggcctttttgtgacggtct-3) or Sr-sg (5-gagacggttgtaaacgtctc-3). The U251 or U87MG cells cultured in a 100 mm dish at about 80% confluence were transfected with the individual sgRNA-Cas9 plasmid using lipofectamine 3000 reagent. Two days after transfection, the cells were passaged at 1:4 ratios into 100 mm culture dishes and 2 days later were treated with 2 μg/ml puromycin. About 96 puromycin-resistant clones were picked up for each gene knockout. Positive clones were selected by immunofluorescent staining and Western blot before directly sequencing of their DNA PCR products to confirm their knockout. The Sr-sgRNA-transfected cells were selected with puromycin and drug-resistant cell pool was used as knockout control, hereafter referred to as wild type (WT).

### Gene knockdown by shRNA lentivirus

Overnight culture of freshly isolated GBM cells from NSG recipients were transduced with high-titer lentiviral mixture of HIF1α-sh1 and HIF1α-sh2 or with scrambled-sh (Sr-sh) controls for 30 h before checking the expression of GFP-reporter under microscope and the 1 × 10^5^ transduced cells/mouse were injected intracranially into NSG mice. The two lentiviral shRNA plasmids were constructed by cloning DNA oligo sequences of HIF-1α (sh1, 5-gcgaagtaaagaatctgaag; sh2, 5-gaaactcaagcaactgtca) or Sr-sh (sh1, 5-gtgctatcacctcactgaa; sh2,5-gacatctcgacgtgcagcaa) into a lentiviral shRNA vector with GFP as reporter [25].

### Promoter and chromatin immunoprecipitation (ChIP) assays

#### Promoter

The proximal promoter regions of human *PDGFD* and *PDGFRA* were PCR amplified from genomic DNA of healthy human PBMCs. Primers used for PCR were as follows: PDGFD, forward (5′-gaaggcaagtgagcacagtgttct-3′) and reverse (5′-agctctccccaaacttcctgcat-3′); *PDGFRA* forward (5′-attgtcatattggactcaacagtt-3′) and reverse (5′-accttctcctccgatgttattc-3′). To establish promoter reporters, the PCR fragment was cloned into lentiviral vector upstream of the GFP reporter to allow promoter-driven expression of GFP. For the *PDGFRA* promoter construct, DNA oligos of the *PDGFRA* enhancers (located within the first intron containing consensus HRE sites) were synthesized and inserted downstream of the PDGFRA proximal promoter. The DNA sequences of the enhancers are as follows: E1 (5′-ctacccacggccgtgcggctctcgtgcccatag-3′), E2 (5′-caacccgtggacgcacgtccttggaccaacactg-3′).

#### ChIP assay

U251 cells were starved of serum for 8 h and re-flashed with medium containing 10% FBS for 2 h before 1% formalin fixation. The rabbit HIF1α antibody was used for immunoprecipitation of sonicated SDS lysate diluted at 1:10 ratio with dilution buffer. The following steps were performed according to the guidelines for ChIP Assay Kit (17–295, Millipore). Control IgG was used as a parallel control. HIF1α-bound regions in the promoters of *PDGFD* and *PDGFRA* pulled down by ChIP were amplified by PCR using primer pairs as follows: *PDGFD*, forward (5′-aggcaagtgagcacagtgttctg-3′) and reverse (5′-taccagagagtattggacacc-3′); *PDGFRA*, proximal promotor P1 forward (5′-cctgacagctatttacttaga-3′) and reverse (5′-cttctcctccgatgttattc-3′); enhancer E1 forward (5′-ctggtctcgaactcctgacct-3′) and reverse (5′-aggggtttagggttacagga-3′); enhancer E2 forward (5′-caactgaggtcaccacgaaag-3′) and reverse (5′-tcctaatggtctccgcgaag-3′).

#### Promoter activity

HEK293T cells were plated onto a 24-well plate (2 × 10^5^ cells per well) 12 h before transfection. The cells were transiently transfected with various promoter plasmids and with/without HIF1α-PPN (P402A/P564A/N803A mutant) plasmid (total DNA 500 ng/per well) using lipofectamine 3000. Twenty-four or 36 h after transfection, the GFP signals of the cells were photographed under fluorescence microscope and the mean fluorescence intensity (MFI) of the cells was determined by flow cytometry. Red fluorescent control plasmid was co-transfected with these promoter plasmids as internal control to read out transfection efficiency of each sample. One of quadruplicate wells was fixed with cold methanol to stain the Flag-tagged HIF1α-PPN to confirm its expression.

### In vivo tumor models

GBM cells, 1 × 10^5^ in 3 μl DMEM medium, were stereotactically injected into the *cerebral* cortex of each anesthetized recipient NSG (for primary GBM) or C57BL/6 (for GL261) mouse at a depth of 2 mm. Mice were randomly grouped (*n* = 5–6/group), and treated with 250 μg/kg Liposomal Echinomycin (LEM) or vehicle via tail vein injection every other day for a total of 4 injections, beginning on day 10 (xenograft mice) or day 7 (GL261). Survival was determined based on the removal criteria to estimate the Kaplan-Meier survival curves. The WT and KO U251 cells were transduced with lentiviruses of GFP-Luciferase reporter and the sorted cultured GFP-positive cells were injected in the cerebral cortex of NSG mice as with the GBM primary cells.

### Clinical samples for gene expression analysis

We analyzed the gene transcript expression of HIF1α and its targets, PDGFs and PDGFRs in GBM samples (*N* = 174) versus low-grade glioma (LGG) samples (*N* = 529) from TCGA (The Cancer Genome Atlas). The Mann-Whitney test was used to determine statistical significance.

## Results

### High HIF1α activity and PDGFs/PDGFRs expression in GBM

**Fig. 1:**
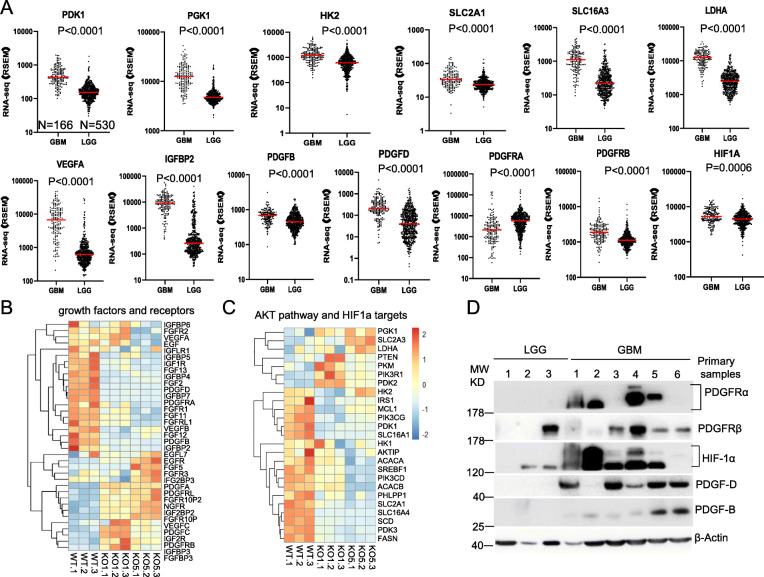
High expression of HIF1α, HIF1α targets, PDGFs and PDGFRα in GBM. **A** Analysis of TCGA database for the mRNA levels of indicated genes in GBM samples (*N* = 174) versus low-grade glioma (LGG) samples (*N* = 529). **B**, **C** HIF1α governs expression of growth factors and receptors, and specific targets and substrates associated with AKT activation. Cas9-guided RNA method was used to generate *HIF1A* knockout (KO) or scrambled sgRNA control (WT) U251 cells for exome RNA-sequencing. Heat-maps depict the differential gene expression profiles among two *HIF1A* KO U251 clones (KO1 and KO5), and WT polyclone control U251 cells, performed in triplicate for each sample. Analysis of growth factors and receptors (B) or HIF1α targets and substrates associated with AKT activation (C) are shown. **D** Immunoblot showing protein levels of indicated genes in freshly frozen tumor tissues from primary surgically-resected adult gliomas

To identify key molecules or pathways in driving GBM malignancy, we analyzed 174 GBM and 529 low-grade glioma (LGG) patient samples from the Cancer Genome Atlas (TCGA). This analysis revealed significantly higher expression of *HIF1α* and its targets in GBM vs LGG, particularly those involved in glycolysis (PDK1, PGK1, HK2, SLC2A1/GLUT1, SLC16A3/MCT4, LDHA), angiogenesis (VEGF-A), and tyrosine receptor signaling pathway (IGFBP2) (Fig. 1A). Growth factors *PDGFA*, *PDGFB* and *PDGFD* were also more highly expressed in GBM (Fig. 1A). Interestingly, expression of *PDGFRA*, a major growth factor receptor in glioma cells, was significantly reduced in GBM, whereas *PDGFRB* was increased (Fig. 1A). To identify which pathways may be controlled by HIF1α in GBM, and to validate our findings from TCGA, we performed exome RNA-sequencing (RNA-Seq) on U251 cells after using Cas9-guided RNA (sgRNA) method to knockout HIF1α. Analysis of growth factors, receptors, and substrates in AKT signaling pathway, and HIF1α target genes involved in glycolysis, de novo lipogenesis, and angiogenesis, revealed that *PDGFD, PDGFRA, IGFBP2, PDK1,3, SLC16A1,3/MCT1,4*, and lipogenesis genes *SCD* and *FASN* were all consistently downregulated in U251 cells following genetic ablation of HIF1α, in comparison to scrambled sgRNA control (Fig. 1B,C, Table S2, S3). These data indicate that the expression of *PDGFRA* and *PDGFD* in GBM cells depends more on HIF1α compared with the expression of other receptors and growth factors, such as *PDGFB*. Moreover, surgically resected primary LGG (*n* = 3) and GBM (*n* = 6) tissue samples were compared for HIF1α, PDGFRα and PDGF-D levels by Western blot. The data revealed high protein levels of HIF1α (5/6), PDGFRα (4/6) and PDGF-D (5/6) in most of the 6 GBM cases (Fig. 1D). These results suggest a positive relationship between HIF1α and PDGF-D and PDGFRα in GBM.

### HIF1α is required for the growth and invasion of GBM cells

**Fig. 2:**
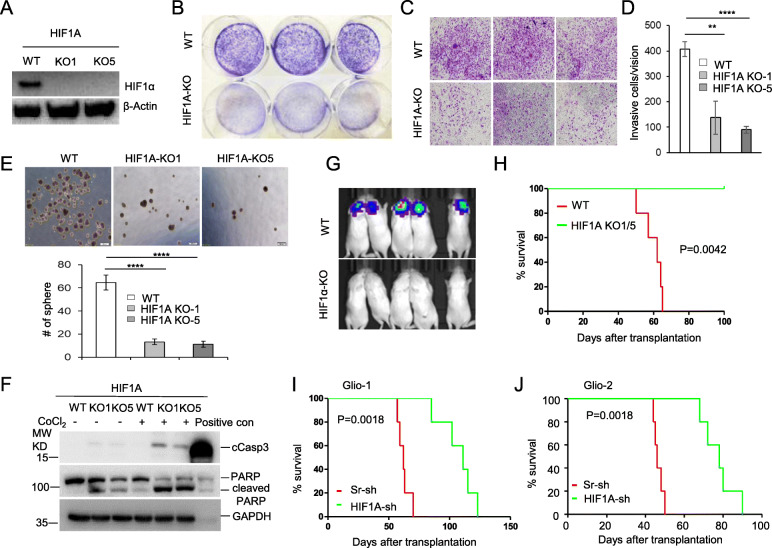
HIF1α is required for the in vitro growth and invasion and the in vivo tumor growth of GBM cells. **A**, **B** Knockout of *HIF1A* retards cell growth. HIF1α protein levels for sgRNA control (WT) or two *HIF1A* KO clones (KO-1, KO-5) are shown by Western blot confirming successful KO (A), and crystal violet staining (B) depicts difference in cell growth among *HIF1A* KO or WT U251 cells; 5 × 10^3^ cells were seeded in a 6-well plate and cultured for 7 days. **C**, **D** *HIF1A* knockout inhibits GBM cell invasion. WT or *HIF1A*-KO U251 cells were seeded in the upper chambers of a 24-well trans-well plate (1 × 10^5^ cells/well) 4–5 days before staining the invasive cells in the lower chambers (C). Number of invasive cells were counted and are shown as average number of cells/vision (D). **E** *HIF1A* knockout inhibits tumor sphere formation. WT and *HIF1A*-KO U251 were seeded in a low-touch 6-well plate (1 × 10^5^cells/well). After 7 days of culture, tumor spheres formed in serum-free NeuroCult culture medium were photographed (E, upper panel) and total number of tumor spheres/well were counted (E, lower panel). **F** *HIF1A* KO U251 cells are prone to apoptosis and sensitive to CoCl_2_-induced apoptosis compared with WT U251 cells. Western blot depicts levels of apoptotic proteins cleaved-caspase 3 (cCasp3) and PARP or cleaved PARP, in WT U251 cells or *HIF1A* KO clones KO1 or KO5, with or without CoCl2 treatment. **G**, **H** *HIF1A* knockout eliminates tumor growth in vivo. NSG mice received orthotopic transplantation of WT or *HIF1A* KO U251 cells (5 × 10^4^ cells/mouse) and bioluminescence imaging was performed. (G). Survival curves for mice that received WT or *HIF1A*-KO cells were estimated within the period of 100-day observation (H). **I**, **J** Knockdown of *HIF1A* by mixed *HIF1A*-sh silencers in primary GBM cells retarded GBM growth in NSG recipients compared with scrambled-sh (Sr-sh) control cells. Survival curves for mice that received high-titer lentivirus-transduced Glio-1 or Glio-2 cells at 5 × 10^4^ cells/mouse were estimated within 100–120 days after intracranial implantation. Error bars represent standard deviation (SD) over three independent experiments, each performed in triplicate. Data shown are representative of three independent experiments

Whether HIF1α plays a critical role in GBM growth remains undefined, although HIF1α knockdown was shown to inhibit growth and invasion of glioma cell lines in vitro [40]. We used sgRNA to target *HIF1A* in GBM cell lines, U251 and U87MG. The two clones with *HIF1A* knockout (KO) in U251 cells were confirmed by Western blot and by directly sequencing their PCR products (Fig. 2A and Fig. S1). Both *HIF1A* KO U251 clones exhibited reduced growth (Fig. 2B) and invasion (Fig. 2C,D). The size of tumor spheres was also reduced (Fig. 2E). In addition, *HIF1A* KO U251 cells cultured at low cell density exhibited increased levels of apoptotic proteins, cleaved caspase 3 (cCasp3) and cleaved PARP (bottom bands). The trend was more pronounced when the cells were treated with the hypoxia mimetic CoCl_2_. More strikingly, *HIF1A* KO U251 clones were unable to either form tumors or cause mortality in xenograft recipient NSG mice (Fig. 2G, H). Similarly, the *HIF1A* mutated clone of U87MG cells also exhibited greatly reduced colony formation and delayed tumor growth in xenograft recipients (Fig. S2B, C).

To test if *HIF1A* is critical for tumor growth in primary GBM cells, we performed similar experiments in xenograft mice using Glio-1 and Glio-2 cells. To maintain the heterogeneity of primary GBM, we used high-titer lentiviral *HIF1A* silencers to infect the cells prior to intracranial transplantation. As shown in Fig. S3A, infection efficiency was similar in *HIF1A* shRNA- or scrambled shRNA- infected cells, based on expression of the GFP reporter. Co-transfection of HEK293FT cells with *HIF1A*-sh-GFP and HIF1α-P2A-RFP plasmids validated the successful shRNA knockdown of *HIF1A* mRNA by the expression of RFP and GFP reporters, as the HIF1α-P2A-RFP plasmid allows for HIF1α and RFP to be expressed as a single mRNA transcript which is then translated as two proteins, separated by the self-cleaving peptide P2A (Fig. S3B). As shown in Fig. 2I and J, silencing *HIF1A* in the Glio-1 or Glio-2 primary cells also restrained tumor growth and significantly extended survival of recipient mice compared to scrambled sh-RNA control cells (Sr-sh) (Fig. 2 I, J). Taken together, these data indicate that HIF1α plays a critical role in the growth and in vitro invasion of GBM cells.

### HIF1α regulates expression of *PDGFRA* and *PDGFD* in GBM cells

**Fig. 3:**
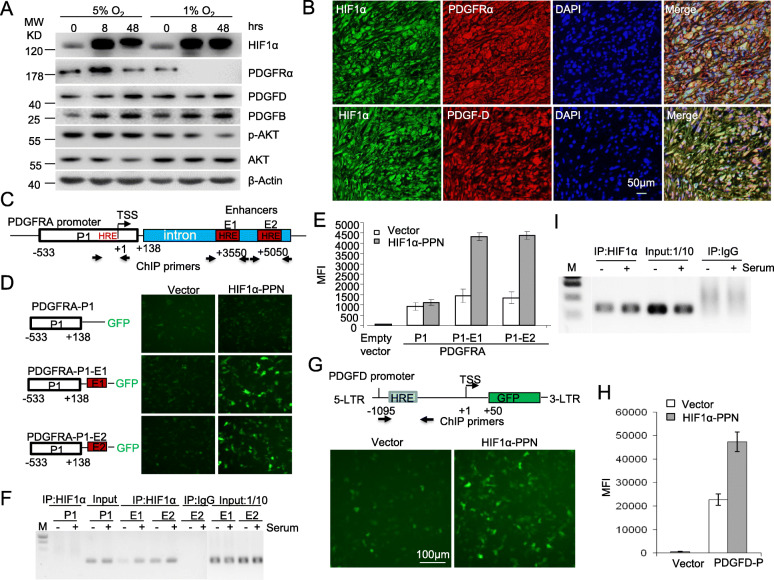
HIF1α regulates the expression of *PDGFRA* and *PDGFD* in GBM cells. **A** WT U251 cells were cultured in the indicated hypoxic conditions and protein expression was detected by Western blot. **B** Co-expression of HIF1α with PDGFRα and PDGF-D in GBM tissue arrays were examined by immunofluorescence co-staining. C-F. HIF1α directly regulates *PDGFRA* promoter activity. **C** Diagram of 5′ region of the *PDGFRA* gene, with positions of three HRE sites, its proximal promoter region, intronic enhancer regions and locations of ChIP primers, is shown. **D** Induction of *PDGFRA* promoter activity by HIF1α. HEK293 cells were co-transfected with plasmids of *PDGFRA* promoter and either empty vector control or HIF1α-PPN. Twenty-four hours after transfection, GFP expression was visualized by fluorescence microscopy, depicted in the representative photographs. PDGFRA-P1 corresponds to the promoter construct containing promoter alone; E1, E2 denote either of the intronic enhancers. **E** GFP mean fluorescence intensity (MFI) was quantitated by flow cytometry and summarized for cells transfected in triplicate. **F** HIF1α binds to the enhancers of *PDGFRA* promoter. ChIP assay was conducted in U251 cells using rabbit HIF1α antibody and rabbit IgG as a parallel control. One tenth of lysate was used as the input and the data shown is representative of 3 experiments. **G**, **H** Induction of *PDGFD* promoter activity by HIF1α was performed as in D,E. **I** HIF1α binds to the proximal HRE site of the *PDGFD* promoter as in F. The SD is from three independent experiments each performed in triplicate

As oxygen availability is unevenly distributed throughout GBM tumors, we examined the impact of differential oxygenation on protein levels of HIF1α, PDGFRα, PDGF-D, and phospho-AKT in WT or *HIF1A*-KO U251 cells exposed to a range of oxygen tensions. As shown in Fig. 3A, in wild type cells, mild hypoxia (5% O_2_, 8 h) increased protein levels of both HIF1α and PDGFRα with minimal effect on PDGF-D and PDGF-B, whereas moderate (5% O_2_, 48 h) or severe (1% O_2_, 48 h) hypoxia increased PDGF-D and PDGF-B protein levels but dramatically reduced that of PDGFRα (Fig. 3A, S3C). However, neither mild nor moderate hypoxia increased the activation of AKT and, like PDGFRα, severe hypoxia actually reduced AKT activation despite maintaining the accumulation of HIF1α (Fig. 3A). *HIF1A* knockout dramatically reduced the expression of both PDGFRα and PDGF-D, moderately reduced PDGF-B, but increased PDGFRβ at normoxia (Fig. S3C). Combined with the growth reduction and hypoxia-induced apoptosis in *HIF1A*-KO cells (Fig. 2), these results indicate that HIF1α favors GBM cell growth in normoxic and mild to moderate hypoxic conditions in which the growth factors and the receptor PDGFRα and the AKT activation are all maintained persistently.

To ascertain the correlation between HIF1α and PDGFRα, we tested expression of HIF1α and PDGFRα among 35 cases of GBM tissues. We observed high expression of both proteins in 21/35 GBM cases, depicted in the representative images (Fig. 3B, upper row), and intensity of HIF-1α staining was highly correlated with that of PDGFRα , with rare instances of single-positive staining (Fig. S4C). In addition to GBM, co-expression of HIF1α and PDGFRα proteins was also observed in other types of gliomas, but not in medulloblastoma or adjacent normal brain tissues (ANB) (Fig. S4 A,B). Similarly, we observed a correlation between HIF1α and PDGF-D expression with double positive stains observed in 23 of 38 cases of a GBM tissue microarray (Fig. S4C), and representative double-positive staining shown in Fig. 3B, bottom row.

To see if HIF1α directly regulates the expression of *PDGFRA* and *PDGFD* in GBM, we first identified a putative hypoxia-response element (HRE) in the *PDGFRA* proximal promoter, and 2 HREs within intron 1 of the *PDGFRA* gene (Fig. 3C). We cloned the *PDGFRA* proximal promoter, with or without its intronic HREs, into a GFP-reporting vector and cotransfected the constructs with mutant HIF1α-PPN (a P402A/P564A/N803A mutant resistant to degradation under normoxia) into HEK293 cells. As shown in Fig. 3D and E, HIF1α did not induce the proximal promoter activity of *PDGFRA* (PDGFRA-P1). Adding either intronic HRE to the basic promoter upstream of the GFP reporter (PDGFRA-P1-E1 and PDGFRA-P1-E2, respectively) resulted in the activation of the *PDGFRA* promoters (Fig. 3D,E). The PDGFRA-P1-E1 promoter activity was also activated by hypoxia mimetic CoCl_2_ in concentrations ranging from 50 to 250 μg/ml (Fig. S3D). Chromatin immunoprecipitation (ChIP) in WT U251 cells with or without 8-h serum-starvation revealed that endogenous HIF1α can bind to the regions encompassing each of the enhancers, but not to the HRE region located in the proximal basic promoter of *PDGFRA* under normoxic conditions (Fig. 3F). These results demonstrated that HIF1α regulates the expression of *PDGFRA* through binding to the *PDGFRA* enhancers rather than its basic promotor.

**Fig. 4:**
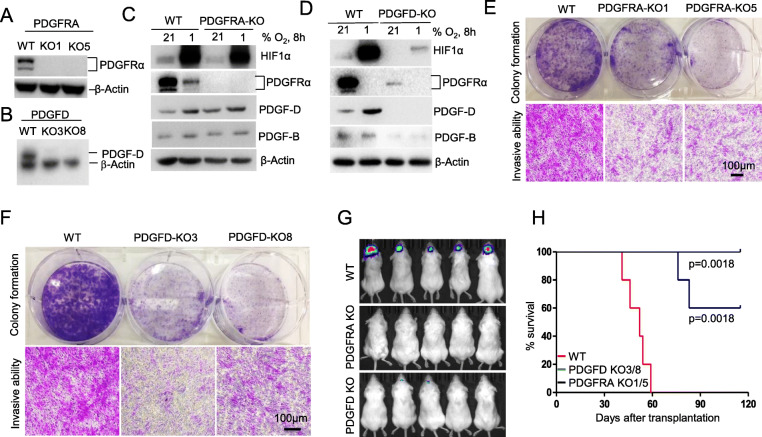
PDGFRα and PDGF-D are essential for GBM growth and invasion. **A**, **B** sgRNA knockout of *PDGFRA* or *PDGFD* in U251 cells was confirmed by Western blot. **C**, **D** *PDGFRA* KO cells and *PDGFD* KO cells were treated with hypoxia and the expression of HIF1α and others was detected by Western blot. **E**,**F** The knockout of *PDGFRA* or *PDGFD* inhibits colony growth and cell invasion compared with scrambled sgRNA polyclonal WT cells. KO or WT cell lines were seeded into a 6-well plate (5 × 10^3^ cells/well) and the reduced growth of the KO cells was determined by colony growth assay (E,F, upper). The KO or WT cell lines were seeded into the upper chamber of a 24-well transwell plate (1 × 10^5^ cells/well) and the cell invasive activity shown in (E,F, lower). **G**, **H** PDGFRα and PDGF-D are essential for tumor growth. WT, PDGFRA-KO and PDGFD-KO cells (mixed clones of each) were transduced with GFP-Luciferase, and intracranially transplanted into NSG mice with 5x10^4^cells/mouse for each line. Kaplan-Meier survival curves are shown for the mice. Three independent experiments were performed to confirm the roles of PDGFRα and PDGF-D in tumor cell growth and invasion and tumor formation in mice

HIF1α also activated the proximal promoter activity of *PDGFD* in HEK293 cells co-transfected with plasmids of stable HIF1α-PPN and the PDGFD promoter (PDGFD-P-GFP) (Fig. 3G). Compared to empty vector, HIF1α enhanced the promoter activity more than 2-fold, as determined by flow-cytometry (Fig. 3H). CoCl_2_ also activated the *PDGFD* promoter activity at concentrations ranging from 100 to 500 μg/ml (Fig. S3D). ChIP revealed endogenous HIF1α bound to the proximal promoter region of *PDGFD* in U251 cells, which was also modestly increased by serum stimulation (Fig. 4I). No specific PCR product could be detected in control IgG ChIP assay, which confirmed the specificity of HIF1α binding to *PDGFD* (Fig. 4I). Therefore, HIF1α directly regulated PDGF-D expression in GBM.

### PDGFRα and PDGF-D are required for invasion and growth of GBM cells

To test if HIF1α-regulated PDGF-D and PDGFRα are essential for GBM growth and invasion, we generated *PDGFRA* KO or *PDGFD* KO U251 cells by Crispr-Cas9 sgRNA editing method (Fig. 4A,B). Knockout of PDGFRA minimally affected HIF1α, PDGF-D and PDGF-B levels, at either normoxic or hypoxic conditions (Fig. 4 C), whereas knockout of *PDGFD* dramatically reduced protein levels of HIF1α, PDGFRα and PDGF-B regardless of oxygen levels (Fig. 4D). Knockout of *PDGFRA* or *PDGFD* dramatically reduced growth and invasion of U251 cells in vitro when cells were seeded at low cell density (Fig. 4E, F). More importantly, these *PDGFRA* KO lines did not grow or cause mortality in NSG recipient mice throughout the observation period of 120 days (Fig. 4G, H). Mice that received *PDGFD* KO lines had significantly longer survival than mice engrafted with *PDGFD* WT cells (Fig. 4H). These results demonstrated that HIF1α controls GBM growth mainly through upregulating the expression of PDGFRα and PDGF-D.

### HIF1α-PDGFD-PDGFRα pathway controls constitutive activation of AKT, leading to GBM cell growth and invasion

**Fig. 5:**
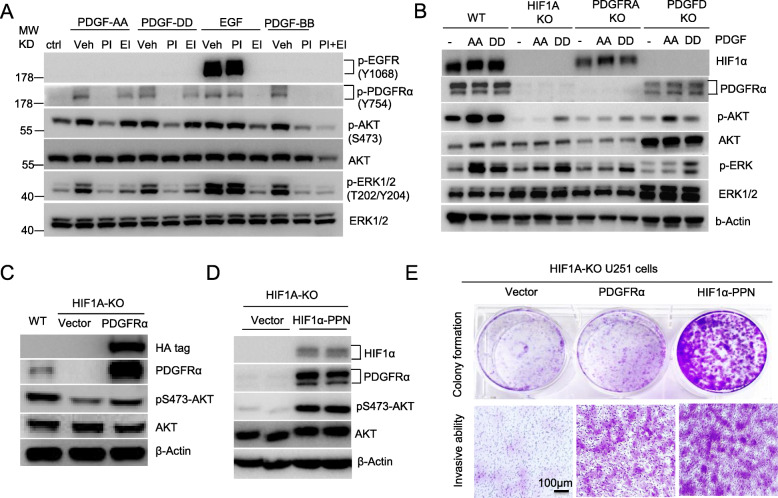
HIF1α-PDGFD-PDGFRα pathway controls constitutive activation of AKT in GBM cells. **A** PDGF-PDGFRα signaling relays the activation of downstream pathways of AKT and ERK independent of EGFR activation. Overnight serum-starved U251 cells were treated with 50 ng/ml each of growth factors as indicated for 10 min in the absence or presence of 30 min pretreatment with 100 ng/ml of PDGFR inhibitor AG1296 (PI), or 100 ng/ml EGFR inhibitor AG1478 (EI), before being lysed for Western blot. **B** HIF1α-PDGF-D-PDGFRα axis is required for a constitutive AKT activation. Serum-starved WT or KO U251 cells were treated with 50 ng/ml of PDGF-AA or PDGF-DD for 10 min before being lysed for Western blot. **C**,**D**,**E** Ectopic expression of PDGFRα (C) or HIF1α (D) in *HIF1A*-KO cells restored AKT activation (C,D) and the cell growth and invasion (E). *HIF1A*-KO U251 cells were transduced with lentiviruses of PDGFRα or HIF1α-PPN to establish stable cells expressing them for Western blot (D) and invasive assay (E). Representative experiments are shown from three independent experiments

**Fig. 6:**
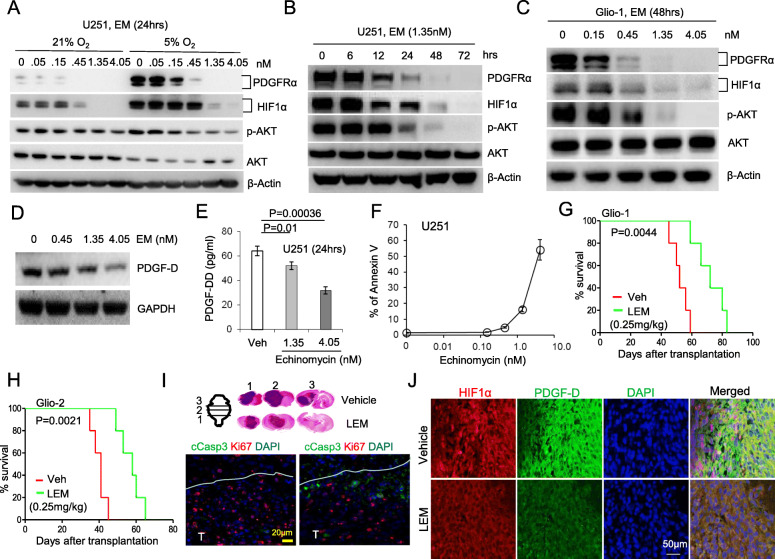
Echinomycin blocks HIF1α-PDGFD-PDGFRα autocrine/feedforward pathway and blunts AKT activation, leading to the apoptosis of GBM cells and the retardation of implanted tumors. **A**,**B**,**C**,**D** Echinomycin blocks HIF1α accumulation and AKT activation and the expression of PDGFRα and PDGF-D. U251 cells (A, B, D) or primary Glio-1 cells (C) were treated with Echinomycin at different dosages and times as indicated, at normoxia or mild hypoxia (A). Indicated protein levels were detected by Western blot. **E** Echinomycin inhibits the release of PDGF-D from U251 cells. The cells were treated with Echinomycin for 24 h before detecting PDGF-DD levels released in the treated medium. **F** Echinomycin induced apoptosis of U251 cells. Annexin V staining was performed on U251 cells that were treated for 48 h with different dosages of Echinomycin. The average percentage of apoptotic cells from three independent experiments was readout by flow cytometry. **G**, **H** Liposomal echinomycin (LEM) improves the survival of primary Glio-1 or Glio-2 bearing NSG mice. Kaplan-Meier survival curves are shown for the recipient mice treated with LEM or vehicle as described in methods. Data shown are representative of three independent experiments. **I** Histological staining of Glio-1 mice. NSG mice bearing Glio-1 tumors were treated with vehicle or LEM as described in methods and perfused for histological staining on day 50. Serial sections were cut for brain tissues spanning the tumor site as diagramed in I, top left. I, top right depicts the H&E stained sections of vehicle or LEM treated mice through each of three serial cuts (numbered 1–3) indicated in the diagram. Immunofluorescence staining depicts the co-staining of cleaved caspase 3 and Ki67 (I, lower). The letter T marks the region of GBM tumor, with white line marking the boundary between tumor and adjacent normal brain tissue. **J** Immunofluorescence staining of Glio-1 mice as in I, showing co-staining for HIF1α and PDGF-D

PDGF-D was reported to bind to and activate PDGFRβ/β homodimer and PDGFRα/β heterodimer in cells expressing both receptors [41, 42]. As PDGFRβ expression was much lower in U251 cells than PDGFRα expression, we compared PDGF-D to other PDGF family members for their ability to activate PDGFRα, and if so, whether such interactions result in the HIF1α accumulation in normoxia. Stimulating U251 cells with recombinant PDGF-A, PDGF-B or PDGF-D induced phosphorylation of PDGFRα to a comparable extent, which was blocked by the PDGFR-specific tyrosine kinase inhibitor AG1296 (Fig. 5A), indicating PDGFRα acts as their receptor. Although the transactivation EGFR by PDGF-B was reported in fibroblasts [43], none of these PDGFs activated the phosphorylation of EGFR in U251 cells (Fig. 5A). The activation of PDGF to PDGFRα relayed signals to its down-stream pathway activation of both AKT and ERK, as the PDGFR inhibitor blocked their phosphorylation completely. The EGFR inhibitor AG1478 at 0.5 μM concentration slightly inhibited the PDGFRα activation, as well as the activation of AKT and ERK induced by PDGF-D, whereas it completely blocked EGF-induced EGFR signaling cascades (Fig. 5A). The PDGFD-PDGFRα-AKT signaling cascade required HIF1α for its constitutive activation because knockout of HIF1α abolished the expression of both the ligand and receptor, and then the phosphorylation of AKT (Fig. 5B). This pathway activation is also required for HIF1α accumulation in normoxia (Fig. 5B) because knockout of either *PDGFD* or *PDGFRA* abolished or reduced HIF1α protein levels. Compared to AKT activation, transfection of *PDGFRA* to *HIF1A* KO U251 cells not only moderately increased AKT activation and cells growth, but also greatly enhanced the cell invasive ability in vitro (Fig. 5 C, E). Overexpression of HIF1α in *HIF1A* KO U251 cells completely restored the PDGFRα expression, AKT activation, and thus the colony growth and cell invasion in vitro (Fig. 6 D, E).

It is worth noting that ERK activation either in the absence of exogenous PDGF or in the presence of exogenous PDGF-D was moderately affected by ablation of *HIF1A*, although its activation by PDGF-A was dramatically affected by the *HIF1A* ablation (Fig. 5B). Since ERK activation is moderately affected by deletion of *PDGFRa*, and since PDGFRβ levels increased in the *HIF1A* knockout cells (Fig. S3C), the basal and PDGF-D-induced activation of ERK may relate to PDGF-D interaction with PDGFRβ or other receptors that are independent of HIF1α and PDGFRα. Nevertheless, ERK activation by PDGFRβ signaling pathway cannot compensate the loss of HIF1α-PDGFD-PDGFRα-AKT network for GBM tumor growth because knockout of either *HIF1A* or *PDGFD* or *PDGFRA* in U251 cells eradicated the tumor growth (Fig. 2G, H and Fig. 4G, H). As *HIF1A* knockout also abolished PDGF-D release (Fig. S5A), and *PDGFD* knockout largely reduced HIF1α protein levels, their reciprocal regulation is both autocrine and feedforward.

### Echinomycin inhibits HIF1α-PDGFD-PDGFRα-AKT signaling and induces apoptosis of GBM cells

Having established the novel feedforward mechanism of HIF1α-PDGFR-AKT pathway, we tested the effect of HIF1α inhibitor Echinomycin in regulating this new pathway. Echinomycin inhibited PDGFRα expression and AKT activation in a dose-dependent manner under normoxic or mild hypoxic conditions (Fig. 6A). The inhibition was also time-dependent, as shown in Fig. 6B. We observed a similar dose-response when echinomycin was used to treat primary Glio-1 cells (Fig. 6C). Figures 6D and E showed that echinomycin also inhibited PDGF-D secretion in a dose-dependent manner from U251 cells. Correspondingly, Echinomycin induced apoptosis of U251 cells in a dose-dependent manner (Fig. 6F and S5B), although it had minimal effects on the viability of *HIF1A* KO cells at low doses when compared with WT cells, confirming its on-target effect (Fig. S5C). Similar to *HIF1A* KO cells, the *PDGFRA* KO cells also exhibited resistance to Echinomycin treatment (Fig. S5D), which suggests that the two proteins work on the same pathway for cell viability.

### Targeting HIF1α by liposomal echinomycin inhibits tumor growth and prolongs survival of GBM-xenografted NSG mice

To test the impact of pharmacologically targeting the HIF1α-PDGFD-PDGFRα axis in GBM in vivo, we took advantage of an improved formulation of Echinomycin which we developed recently using liposomes (LEM) to treat solid tumors [44, 45]. LEM prolonged survival in mice xenografted with primary Glio-1 or Glio-2 tumors by about 20 days (Fig. 6G,H). By sequencing the PCR products of the genomic DNA encompassing exons 2–9 of the *TP53* gene, we found that Glio-1 cells were surprisingly contained the same R273H hot-spot mutation as U251 cells, albeit heterozygous, whereas no *TP53* mutation was observed in Glio-2 (Fig. S5E). However, regardless of TP53 mutation status, LEM conferred similar survival advantages to mice engrafted with either Glio-1 or Glio-2, indicating that HIF1α and HIF1α-controlled PDGFD-PDGFRα-AKT signaling contributes more to the sensitivity to Echinomycin treatment. Indeed, in comparison to vehicle controls, Echinomycin inhibited the Glio-1 tumor growth as seen in the reduced tumor size of representative sections (Fig. 6I, top), and the proliferation as judged by the reduced proliferative marker Ki67 in contrast to the increased apoptotic marker cleaved-caspase3 (Fig. 6I, bottom). Echinomycin also reduced immunofluorescent staining of HIF1α and PDGF-D in GBM tissues (Fig. 6J). These results demonstrate that Echinomycin effectively targets the HIF1α-PDGF-D axis to inhibit GBM growth.

### Overexpression of PDGF-D stimulates tumor growth and angiogenesis in immunocompetent mice, and renders sensitivity to echinomycin treatment

**Fig. 7:**
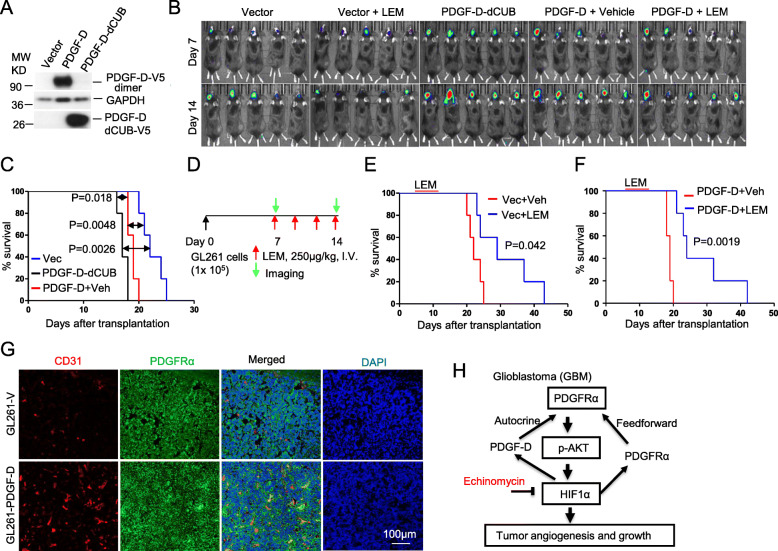
Overexpression of PDGF-D increases angiogenesis and sensitivity to LEM treatment in immunocompetent mice. **A** Anti-V5 tag antibody detected the ectopic expression of PDGF-D or PDGF-D-dCUB in the mouse GBM line GL261 expressing luciferase. **B**-**F** C57BL/6 were orthotopically transplanted with GL261 cells lentiviral-transduced by vector control, PDGF-D-dCUB, or PDGF-D, as indicated in the headings, and treatment with vehicle or LEM was administered as described in methods. Bioluminescence imaging is shown for the different groups (B). The survival of mice receiving vector-, PDGF-D-, or PDGF-D-dCUB-transduced GL261 cells is shown in (C). Experimental design and treatment schedule with LEM is depicted in (D), and the Kaplan-Meier survival curves of mice receiving vector- or PDGF-D-transduced GL261 are shown in (E) and (F), respectively, comparing survival among vehicle or LEM-treated groups. **G** Representative immunofluorescent staining of vector- (GL261-V) or PDGF-D-transduced (GL261-PDGF-D) brain tumor tissue sections are shown from PBS-perfused recipient mice after staining for CD31 or PDGFRα. **H** Summary of the underlying mechanism

Unlike PDGF-B, which is expressed and released as an active homodimer, PDGF-D is expressed and released as an inactive homodimer that is activated by extracellular serine proteinases [46]. Thus, it has been unclear whether PDGF-D played a significant role in GBM pathogenesis. In murine GL261 GBM cells, ectopic overexpression of PDGF-D (Fig. 7A) significantly accelerated tumor growth and mortality of recipient mice (Fig. 7B,C). Moreover, overexpression of the active form of PDGF-D (i.e. PDGFD-dCUB), resulting from deletion of the inhibitory CUB domain (Fig. 7A) [46] had an even more pronounced effect (Fig. 7B,C). The data indicates that GBM tumors are capable of proteolytically activating the potent growth factor PDGF-D. In response to a cycle of treatment (Fig. 7D), Echinomycin effectively neutralized the growth advantage of PDGFD-transduced GL261 tumors and conferred equivalent therapeutic effects for both transduced and un-transduced GL261 tumors (Fig. 7E,F). Immunofluorescence of PDGFRα and angiogenic marker CD31 in tumor tissues revealed an increase in GL261-PDGF-D vs GL261-vector tissues (Fig. 7G). LEM treatment of the GL261 brain tumor greatly reduced the expression of *Pdgfd*, *Pdgfra* and *Igfbp2*, as detected by quantitative RT-PCR, compared to vehicle control tumors (Fig. S6).

Taken together, we demonstrate that HIF1α is a crucial effector on the constitutive activation of AKT through controlling the expression of PDGF-D and PDGFRα in an autocrine and feedforward manner for GBM growth and malignancy, which could be therapeutically targeted by LEM (Fig. 7H).

## Discussion

GBM is characterized by cellular heterogeneity, integrated oncogenic signaling pathways, intratumorally-intricate microenvironments, and distinct regions including a necrotic/hypoxic core surrounded by intermediate/hypoxic layer and by highly proliferative, well-oxygenated and -vascularized outer layer/frontier [27, 28]. Collectively, these complex features of GBM undermine therapeutic strategies targeting single pathways in isolation [47, 48]. HIF1α expression pattern was heterogenous seen in the hypoxic zone and the normoxic frontier of GBM [49]. Our studies revealed that, to different extents, HIF1α ablation reduced constitutive activation of both AKT and ERK signaling pathways in U251 cells. Thus, HIF1α may function as a converging point between these signaling pathways via controlling the expression of PDGF-B, PDGF-D and PDGFRα for the malignancy of GBMs. In addition, based on RNA-Seq data of U251 cells, HIF1α may also control IGF-IGF1R-AKT and FGF signaling pathways for GBM growth and invasion. Thus, HIF1α is a crucial master transcriptional factor that orchestrates the expression of growth factors, receptors, signal-pathway substrates, and angiogenic factors under conditions of normoxia and physiological hypoxia. Inhibiting or deleting HIF1α greatly restrains growth advantages exploited by GBM and may disrupt the reciprocal or feedback regulation between PDGFRα-PI3K-AKT and EGFR-ERK pathways once targeting them separately [50]. Meanwhile, targeting HIF1α could largely block angiogenesis via inhibiting expression of VEGF, PDGF-B and PDGF-D; the latter two of which may promote angiogenesis through binding VEGFR2 and coreceptor neuropilin 1, respectively [51, 52]. Taken together, these features provide a rationale for supporting development of HIF1α-targeting therapies, such as echinomycin, for the treatment of GBM.

HIF1α is normally degraded under conditions of normoxia, although noncanonical mechanisms are known to promote HIF1α stability regardless of oxygen tension. Our studies provide additional evidence for noncanonical HIF1α stabilization, as PDGFD and PDGFRα are both required for high HIF1α accumulation in GBM cells under normoxia. While PDGFD was found to be a hypoxia-induced gene in our study, expression of PDGFRA was only induced by mild hypoxia or normoxia, and AKT activation was only induced under normoxia in GBM cells. Therefore, the paracrine activity of PDGF-B and PDGF-D originating from intermediate/hypoxic middle-layer of GBM via canonical HIF1α stabilization may trigger the HIF1α stabilization in the GBM cells located in the oxygen-rich leading edge of the tumor. More importantly, as PDGFD, PDGFRα, and HIF1α were all required for GBM cell growth and invasion under normoxia and in xenografted mice, our work implies that the PDGFD-PDGFRα-HIF1α axis could be an essential event for GBM malignancy, which is predominantly functional under normoxic or mildly hypoxic conditions. The HIF1α driven *PDGFD* and *PDGFRA* transcription in these conditions suggest that HIF1α promotes a feedforward PDGFD-PDGFRα-AKT signaling in the GBM leading edge. Thus, our findings provide mechanistic insight as to how GBM invades to surrounding tissues.

PDGF-A stimulation or overexpression and PDGF-B overexpression in glial or neural progenitors of mice have been demonstrated each acting a driver for glioma-like neoplasm or glioma-genesis in those mouse models [53–55]. However, their roles in the growth and malignancy of human glioma cells including GBM cell lines have not been revealed. We showed here that PDGF-D is a potent growth factor for both human and mouse GBM cells. Whether it is able to initiate glioma-like brain tumors in mice remains to be defined.

PDGF and PDGFR are frequently co-expressed in human glioma cell lines as well as high-grade gliomas [28, 31]. Previous study showed that *PDGFB* promoter was activated in breast cancer cells by HIF1α under hypoxia [56]. Consistently, we showed here that HIF1α induced PDGF-B, as well as PDGF-D, in U251 cells under hypoxia. In addition, our data showed that PDGF-D was unexpectedly as potent as PDGF-A and PDGF-B in U251 cells which predominantly expressed PDGFRα, suggesting that PDGF-D may activate PDGFRα via another receptor rather than PDGFRβ. These findings provide important new insights on how PDGF-D promotes GBM pathogenesis.

TP53 mutation is observed in up to 54% of proneural GBM, a subtype which also displays frequent overexpression of PDGFRα [5]. Mutant P53 often drives chemotherapy resistance to the first-line drug temozolomide and is associated with poor prognosis [57, 58]. Targeting HIF1α by liposomal Echinomycin could exert significant therapeutic effects in mouse models of GBM regardless of *TP53* mutations. This effectiveness of Echinomycin is consistent with our previous observation that Echinomycin is effective against TP53 mutated AML [59].

## Conclusions

This report is the first to reveal PDGF-D as a potent growth factor for human GBM cells. HIF1α plays a critical role in constitutive activation of the AKT signaling pathway for GBM growth by controlling expression of PDGF-D and PDGFRα under normoxia and physiological hypoxia. The HIF1α inhibitor Echinomycin abolishes the HIF1α-PDGFD-PDGFRα feedforward axis for constitutive activation of AKT pathway and therefore provides a novel and potentially effective therapeutic approach for GBM.

### Statistical analysis

All statistical analysis was performed using the SPSS 21.0 statistical software program. Quantitative data were expressed as mean ± SD. Comparison of two groups was analyzed with student’s T test. Survival between groups was compared with the log-rank test. A value of *p* < 0.05 was considered statistically significant.

## Supplementary Information

**Additional file 1: Figure S1.** Sequencing of PCR products of genomic DNAs of HIF1A knocked out in U251 cells by HIF1A sgRNA. **Figure S2.** HIF1A KO in U87MG cells inhibits cell growth. A, B. HIF1A KO U87 cells grow slower than WT cells. HIF1A in U87MG cells was knocked out by Cas9-sgRNA method, which was identified by DNA sequencing of PCR product and by Western-blot (A). WT and HIF1A KO U87MG cells were seeded 1 × 10^4^ /well in a 6-well plate and cultured for 5 days prior to crystal violet staining to observe colony growth (B). C. NSG mice implanted with HIF1A KO U87MG cells survive longer than WT U251 cells. NSG mice were intracranially implanted with HIF1A KO or WT U87MG cells (5 × 10^4^/mouse) and Kaplan-Meier survival curves were estimated. **Figure S3.** HIF1α is required for the expression of PDGFD and PDGFRA under normoxia and mild hypoxia. A. The efficiency of lentiviral infection of primary Glio-1 and Glio-2. Freshly isolated cells from tumor recipients were transduced with high titer lentivirus for *HIF1A*-sh (mixture of *HIF1A*-sh1 plus *HIF1A*-sh2) or scrambled control (Sr-sh) by centrifugation at 2500 rpm for 2 h, and were then cultured for additional 30 h before given to mice. The GFP expression in the sh construct was photographed 24 h after transduction and represents transfection efficiency. B. Silencing efficiency of *HIF1A*-sh was determined by fluorescence microscopy of HEK293FT cells 24 h after co-transfection with either *HIF1A*-sh plus HIF1α-P2A-RFP, or Sr-sh plus HIF1α-P2A-RFP as a parallel control. C. Western blots are shown for U251 WT and *HIF1A*-KO cells incubated in normoxic or severe hypoxic incubator for 8 or 48 h before being lysed for Western blot. D. CoCl_2_ activated the promoter activities of PDGFRA and PDGFD. HEK293FT cells were transfected with PDGFRA or PDGFD promoter GFP reporter plasmids for 8 h, then treated with CoCl_2_ for an additional 16 h. GFP expression was then visualized by fluorescence microscopy as shown in the top panels, and quantitated by flow cytometry, summarized below. Bar graphs represent mean fluorescence intensity (MFI) readings ± SD for triplicate wells at each dose. **Figure S4.** HIF1α, PDGFRα and PDGF-D are frequently co-expressed in GBM. A. Co-expression of HIF1α and PDGFRα in GBM. Microarray of brain tumor with adjacent normal tissues was co-stained with primary rabbit HIF1α antibody and mouse PDGFRα antibody, and with secondary antibodies goat anti-rabbit Alexa-Fluor 594 and goat anti-mouse Alexa-Fluor 488 after washing away primary antibodies. DAPI was used to visualize nuclei (blue). B. Summary of microarray cases with double positive staining for HIF1α and PDGFRα, which was presented as percentage of total tumor subtype cases. ANB, adjacent normal brain tissue; OD, oligodendrocytoma; OA, oligoastrocytoma; AA, anaplastic astrocytoma; MB, medulloblastoma; EP, ependymoma. Data shown are representative of two independent experiments. C. Correlation of staining intensity for HIF1α with PDGFRα in GBM tissue microarray. IF staining of a 35 cases tissue microarray of GBM and 5 cancer adjacent normal cerebral tissue was performed with PDGFRα and HIF1α antibodies together as described in A. The double positive stains of cases with low, moderate, high, or very high scores were analyzed for the correlation of HIF1α with PDGFRα. D. Co-expression of HIF1α and PDGF-D in GBM. Sections of brain tumor with adjacent normal brain tissue microarray were co-stained with primary rabbit HIF1α antibody and goat PDGF-D antibody, and with secondary antibodies donkey anti-rabbit Alexa-Fluor 488 and donkey anti-goat Alexa-Fluor 594 after washing away primary antibodies. DAPI was used to visualize nuclei (blue). The correlation of staining intensity for HIF1α and PDGF-D are shown. E. Correlation of staining intensity for HIF1α with PDGFD in GBM tissue microarray. IF staining of a 38-cases tissue microarray of GBM and 6 of normal cerebrum tissues was performed with PDGF-D and HIF1α antibodies as described in D. The double positive stains of 23/38 cases with low, moderate, high, or very high scores were analyzed for the correlation of HIF1α with PDGF-D. **Figure S5.** TP53 mutation did not affect GBM response to Echinomycin. A. PDGF-DD levels in the medium of *HIF1A* KO or WT U251 cells. Levels of released PDGF-D protein were measured by ELISA. B. Echinomycin induced apoptosis of U251 cells. Annexin V staining was performed on U251 cells that were treated for 48 h with different concentrations of Echinomycin. C, D. *HIF1A*-KO or *PDGFRA*-KO cells are resistant to Echinomycin. WT, *HIF1A*-KO or *PDGFRA*-KO U251 cells were treated with different concentrations of Echinomycin for 72 h prior to determining cell viability by MTT assay. E. Primary Glio-1 cells have an R273H mutation of TP53 which is also carried by U251 cells. Sequence chromatograms are shown with arrows indicating R273H mutation. **Figure S6.** Echinomycin reduced the expression of HIF1α target genes. Empty vector-transfected GL261 brain tumor cells were orthotopically transplanted to recipient mice, and the mice were treated with vehicle or LEM as detailed in methods. Twenty-four hours after the final dose, the tumor cells were isolated and the cDNA was used to perform qRT-PCR. **Table S1.** Characteristics of Clinical Glioma Samples. **Table S2.** RNA-Seq data of growth factor related genes expressed in WT and HIF1α KO in U251 cells. **Table S3.** RNA-Seq data of metabolism related genes expressed in WT and HIF1α KO in U251 cells.

## Data Availability

No applicable.

## Abbreviations

ANB: Adjacent normal brain tissues
cCasp3: Cleaved caspase 3
ChIP: Chromatin immunoprecipitation assay
CNS: Central nervous system
EGFR: Epithelial growth factor receptor
ERK: Extracellular regulated protein kinases
FASN: Fatty acid synthase
GAPDH: Glyceraldehyde-3-phosphate dehydrogenase
GBM: Glioblastoma multiforme
GLUT1: Glucose transporter type 1
HGG: High-grade glioma
HIF1α: Hypoxia inducible factor subunit alpha
HK2: Hexokinase 2
HRE: Hypoxia-response element
IGFBP2: Insulin like growth factor binding protein 2
LDHA: Lactate dehydrogenase A
LEM: Liposomal Echinomycin
LGG: Low-grade glioma
MCT4: Monocarboxylate transporter 4
NSG: NOD-SCID IL-2receptor gamma null
PARP: Poly(ADP-Ribose) polymerase
PDGF: Platelet-derived growth factor
PDGF-A: Platelet-derived growth factor A
PDGF-B: Platelet-derived growth factor B
PDGF-C: Platelet-derived growth factor C
PDGF-D: Platelet-derived growth factor D
PDGFRα: Platelet-derived growth factor receptor subunit alpha
PDGFRβ: Platelet-derived growth factor receptor subunit beta
PDK1: Pyruvate dehydrogenase kinase 1
PGK1: Phosphoglycerate kinase 1
RTK: Receptor tyrosine kinase
SCD: Stearoyl-CoA desaturase
sgRNA: Small guide RNA
TGCA: The cancer genome atlas
VEGF-A: Vascular endothelial growth factor A

## References

[1] Thakkar JP, Dolecek TA, Horbinski C, Ostrom QT, Lightner DD, Barnholtz-Sloan JS (2014). Epidemiologic and molecular prognostic review of glioblastoma. Cancer Epidemiol Biomark Prev.

[2] Louis DN, Perry A, Reifenberger G, von Deimling A, Figarella-Branger D, Cavenee WK (2016). The 2016 World Health Organization classification of tumors of the central nervous system: a summary. Acta Neuropathol.

[3] Furnari FB, Fenton T, Bachoo RM, Mukasa A, Stommel JM, Stegh A (2007). Malignant astrocytic glioma: genetics, biology, and paths to treatment. Genes Dev.

[4] Ohgaki H, Dessen P, Jourde B, Horstmann S, Nishikawa T, Di Patre PL (2004). Genetic pathways to glioblastoma: a population-based study. Cancer Res.

[5] Verhaak RG, Hoadley KA, Purdom E, Wang V, Qi Y, Wilkerson MD (2010). Integrated genomic analysis identifies clinically relevant subtypes of glioblastoma characterized by abnormalities in PDGFRA, IDH1, EGFR, and NF1. Cancer Cell.

[6] Cancer Genome Atlas Research N (2008). Comprehensive genomic characterization defines human glioblastoma genes and core pathways. Nature.

[7] Parsons DW, Jones S, Zhang X, Lin JC, Leary RJ, Angenendt P (2008). An integrated genomic analysis of human glioblastoma multiforme. Science.

[8] Brennan CW, Verhaak RG, McKenna A, Campos B, Noushmehr H, Salama SR (2013). The somatic genomic landscape of glioblastoma. Cell.

[9] Lopez-Gines C, Gil-Benso R, Ferrer-Luna R, Benito R, Serna E, Gonzalez-Darder J (2010). New pattern of EGFR amplification in glioblastoma and the relationship of gene copy number with gene expression profile. Mod Pathol.

[10] Koschmann C, Zamler D, MacKay A, Robinson D, Wu YM, Doherty R (2016). Characterizing and targeting PDGFRA alterations in pediatric high-grade glioma. Oncotarget.

[11] Szerlip NJ, Pedraza A, Chakravarty D, Azim M, McGuire J, Fang Y (2012). Intratumoral heterogeneity of receptor tyrosine kinases EGFR and PDGFRA amplification in glioblastoma defines subpopulations with distinct growth factor response. Proc Natl Acad Sci U S A.

[12] Ozawa T, Brennan CW, Wang L, Squatrito M, Sasayama T, Nakada M (2010). PDGFRA gene rearrangements are frequent genetic events in PDGFRA-amplified glioblastomas. Genes Dev.

[13] Puget S, Philippe C, Bax DA, Job B, Varlet P, Junier MP (2012). Mesenchymal transition and PDGFRA amplification/mutation are key distinct oncogenic events in pediatric diffuse intrinsic pontine gliomas. PLoS One.

[14] Paugh BS, Zhu X, Qu C, Endersby R, Diaz AK, Zhang J (2013). Novel oncogenic PDGFRA mutations in pediatric high-grade gliomas. Cancer Res.

[15] Snuderl M, Fazlollahi L, Le LP, Nitta M, Zhelyazkova BH, Davidson CJ (2011). Mosaic amplification of multiple receptor tyrosine kinase genes in glioblastoma. Cancer Cell.

[16] Knobbe CB, Reifenberger G (2003). Genetic alterations and aberrant expression of genes related to the phosphatidyl-inositol-3′-kinase/protein kinase B (Akt) signal transduction pathway in glioblastomas. Brain Pathol.

[17] Stommel JM, Kimmelman AC, Ying H, Nabioullin R, Ponugoti AH, Wiedemeyer R (2007). Coactivation of receptor tyrosine kinases affects the response of tumor cells to targeted therapies. Science.

[18] Little SE, Popov S, Jury A, Bax DA, Doey L, Al-Sarraj S (2012). Receptor tyrosine kinase genes amplified in glioblastoma exhibit a mutual exclusivity in variable proportions reflective of individual tumor heterogeneity. Cancer Res.

[19] Chakravarty D, Pedraza AM, Cotari J, Liu AH, Punko D, Kokroo A (2017). EGFR and PDGFRA co-expression and heterodimerization in glioblastoma tumor sphere lines. Sci Rep.

[20] Eskilsson E, Rosland GV, Talasila KM, Knappskog S, Keunen O, Sottoriva A (2016). EGFRvIII mutations can emerge as late and heterogenous events in glioblastoma development and promote angiogenesis through Src activation. Neuro-Oncology.

[21] Katanasaka Y, Kodera Y, Kitamura Y, Morimoto T, Tamura T, Koizumi F (2013). Epidermal growth factor receptor variant type III markedly accelerates angiogenesis and tumor growth via inducing c-myc mediated angiopoietin-like 4 expression in malignant glioma. Mol Cancer.

[22] Ivan M, Kondo K, Yang H, Kim W, Valiando J, Ohh M (2001). HIFalpha targeted for VHL-mediated destruction by proline hydroxylation: implications for O2 sensing. Science.

[23] Jaakkola P, Mole DR, Tian YM, Wilson MI, Gielbert J, Gaskell SJ (2001). Targeting of HIF-alpha to the von Hippel-Lindau ubiquitylation complex by O2-regulated prolyl hydroxylation. Science.

[24] Yu F, White SB, Zhao Q, Lee FS (2001). HIF-1alpha binding to VHL is regulated by stimulus-sensitive proline hydroxylation. Proc Natl Acad Sci U S A.

[25] Wang Y, Liu Y, Malek SN, Zheng P, Liu Y (2011). Targeting HIF1alpha eliminates cancer stem cells in hematological malignancies. Cell Stem Cell.

[26] Wang Y, Liu Y, Tang F, Bernot KM, Schore R, Marcucci G (2014). Echinomycin protects mice against relapsed acute myeloid leukemia without adverse effect on hematopoietic stem cells. Blood.

[27] Brat DJ, Castellano-Sanchez AA, Hunter SB, Pecot M, Cohen C, Hammond EH (2004). Pseudopalisades in glioblastoma are hypoxic, express extracellular matrix proteases, and are formed by an actively migrating cell population. Cancer Res.

[28] Cantanhede IG, de Oliveira JRM (2017). PDGF family expression in glioblastoma Multiforme: data compilation from ivy glioblastoma atlas project database. Sci Rep.

[29] Li Z, Bao S, Wu Q, Wang H, Eyler C, Sathornsumetee S (2009). Hypoxia-inducible factors regulate tumorigenic capacity of glioma stem cells. Cancer Cell.

[30] Jackson EL, Garcia-Verdugo JM, Gil-Perotin S, Roy M, Quinones-Hinojosa A, VandenBerg S (2006). PDGFR alpha-positive B cells are neural stem cells in the adult SVZ that form glioma-like growths in response to increased PDGF signaling. Neuron.

[31] Nazarenko I, Hede SM, He X, Hedren A, Thompson J, Lindstrom MS (2012). PDGF and PDGF receptors in glioma. Ups J Med Sci.

[32] Pringle NP, Mudhar HS, Collarini EJ, Richardson WD (1992). PDGF receptors in the rat CNS: during late neurogenesis, PDGF alpha-receptor expression appears to be restricted to glial cells of the oligodendrocyte lineage. Development.

[33] Liu KW, Feng H, Bachoo R, Kazlauskas A, Smith EM, Symes K (2011). SHP-2/PTPN11 mediates gliomagenesis driven by PDGFRA and INK4A/ARF aberrations in mice and humans. J Clin Invest.

[34] Hermanson M, Funa K, Hartman M, Claesson-Welsh L, Heldin CH, Westermark B (1992). Platelet-derived growth factor and its receptors in human glioma tissue: expression of messenger RNA and protein suggests the presence of autocrine and paracrine loops. Cancer Res.

[35] Wallmann T, Zhang XM, Wallerius M, Bolin S, Joly AL, Sobocki C (2018). Microglia induce PDGFRB expression in Glioma cells to enhance their migratory capacity. iScience.

[36] Li H, Fredriksson L, Li X, Eriksson U (2003). PDGF-D is a potent transforming and angiogenic growth factor. Oncogene.

[37] LaRochelle WJ, Jeffers M, Corvalan JR, Jia XC, Feng X, Vanegas S (2002). Platelet-derived growth factor D: tumorigenicity in mice and dysregulated expression in human cancer. Cancer Res.

[38] Tchougounova E, Kastemar M, Brasater D, Holland EC, Westermark B, Uhrbom L (2007). Loss of Arf causes tumor progression of PDGFB-induced oligodendroglioma. Oncogene.

[39] Hede SM, Hansson I, Afink GB, Eriksson A, Nazarenko I, Andrae J (2009). GFAP promoter driven transgenic expression of PDGFB in the mouse brain leads to glioblastoma in a Trp53 null background. Glia.

[40] Mendez O, Zavadil J, Esencay M, Lukyanov Y, Santovasi D, Wang SC (2010). Knock down of HIF-1alpha in glioma cells reduces migration in vitro and invasion in vivo and impairs their ability to form tumor spheres. Mol Cancer.

[41] Bergsten E, Uutela M, Li X, Pietras K, Ostman A, Heldin CH (2001). PDGF-D is a specific, protease-activated ligand for the PDGF beta-receptor. Nat Cell Biol.

[42] LaRochelle WJ, Jeffers M, McDonald WF, Chillakuru RA, Giese NA, Lokker NA (2001). PDGF-D, a new protease-activated growth factor. Nat Cell Biol.

[43] Saito Y, Haendeler J, Hojo Y, Yamamoto K, Berk BC (2001). Receptor heterodimerization: essential mechanism for platelet-derived growth factor-induced epidermal growth factor receptor transactivation. Mol Cell Biol.

[44] Bailey CM, Liu Y, Peng G, Zhang H, He M, Sun D (2020). Liposomal formulation of HIF-1alpha inhibitor echinomycin eliminates established metastases of triple-negative breast cancer. Nanomedicine.

[45] Liu Y, Nelson MV, Bailey C, Zhang P, Zheng P, Dome JS (2021). Targeting the HIF-1alpha-IGFBP2 axis therapeutically reduces IGF1-AKT signaling and blocks the growth and metastasis of relapsed anaplastic Wilms tumor. Oncogene.

[46] Huang W, Kim HR (2015). Dynamic regulation of platelet-derived growth factor D (PDGF-D) activity and extracellular spatial distribution by matriptase-mediated proteolysis. J Biol Chem.

[47] Monteiro AR, Hill R, Pilkington GJ, Madureira PA. The role of hypoxia in glioblastoma invasion. Cells. 2017;6(4). 10.3390/cells6040045. 10.3390/cells6040045 PMC5755503 29165393

[48] Alexandru O, Horescu C, Sevastre AS, Cioc CE, Baloi C, Oprita A (2020). Receptor tyrosine kinase targeting in glioblastoma: performance, limitations and future approaches. Contemp Oncol (Pozn).

[49] Zagzag D, Zhong H, Scalzitti JM, Laughner E, Simons JW, Semenza GL (2000). Expression of hypoxia-inducible factor 1alpha in brain tumors: association with angiogenesis, invasion, and progression. Cancer.

[50] McNeill RS, Canoutas DA, Stuhlmiller TJ, Dhruv HD, Irvin DM, Bash RE (2017). Combination therapy with potent PI3K and MAPK inhibitors overcomes adaptive kinome resistance to single agents in preclinical models of glioblastoma. Neuro-Oncology.

[51] Mamer SB, Chen S, Weddell JC, Palasz A, Wittenkeller A, Kumar M (2017). Discovery of high-affinity PDGF-VEGFR interactions: redefining RTK dynamics. Sci Rep.

[52] Muhl L, Folestad EB, Gladh H, Wang Y, Moessinger C, Jakobsson L (2017). Neuropilin 1 binds PDGF-D and is a co-receptor in PDGF-D-PDGFRbeta signaling. J Cell Sci.

[53] Bohm AK, DePetro J, Binding CE, Gerber A, Chahley N, Berger ND (2020). In vitro modeling of glioblastoma initiation using PDGF-AA and p53-null neural progenitors. Neuro-Oncology.

[54] Rahme GJ, Luikart BW, Cheng C, Israel MA (2018). A recombinant lentiviral PDGF-driven mouse model of proneural glioblastoma. Neuro-Oncology.

[55] Westermark B (2014). Platelet-derived growth factor in glioblastoma-driver or biomarker?. Ups J Med Sci.

[56] Schito L, Rey S, Tafani M, Zhang H, Wong CC, Russo A (2012). Hypoxia-inducible factor 1-dependent expression of platelet-derived growth factor B promotes lymphatic metastasis of hypoxic breast cancer cells. Proc Natl Acad Sci U S A.

[57] Muller PA, Vousden KH (2014). Mutant p53 in cancer: new functions and therapeutic opportunities. Cancer Cell.

[58] Wang X, Chen JX, Liu JP, You C, Liu YH, Mao Q (2014). Gain of function of mutant TP53 in glioblastoma: prognosis and response to temozolomide. Ann Surg Oncol.

[59] Wang Y, Liu Y, Bailey C, Zhang H, He M, Sun D (2020). Therapeutic targeting of TP53-mutated acute myeloid leukemia by inhibiting HIF-1alpha with echinomycin. Oncogene.

