# Supplementary material for: The HIF1α-PDGFD-PDGFRα axis controls glioblastoma growth at normoxia/mild-hypoxia and confers sensitivity to targeted therapy by echinomycin
Source: J Exp Clin Cancer Res. 2021 Sep 1;40:278. doi: 10.1186/s13046-021-02082-7 (PMC8411541; doi:10.1186/s13046-021-02082-7)
Supplement: Supplementary file 1 — Additional file 1: Figure S1. Sequencing of PCR products of genomic DNAs of HIF1A knocked out in U251 cells by HIF1A sgRNA. Figure S2. HIF1A KO in U87MG cells inhibits cell growth. A, B. HIF1A KO U87 cells grow slower than WT cells. HIF1A in U87MG cells was knocked out by Cas9-sgRNA method, which was identified by DNA sequencing of PCR product and by Western-blot (A). WT and HIF1A KO U87MG cells were seeded 1 × 10^4^ /well in a 6-well plate and cultured for 5 days prior to crystal violet staining to observe colony growth (B). C. NSG mice implanted with HIF1A KO U87MG cells survive longer than WT U251 cells. NSG mice were intracranially implanted with HIF1A KO or WT U87MG cells (5 × 10^4^/mouse) and Kaplan-Meier survival curves were estimated. Figure S3. HIF1α is required for the expression of PDGFD and PDGFRA under normoxia and mild hypoxia. A. The efficiency of lentiviral infection of primary Glio-1 and Glio-2. Freshly isolated cells from tumor recipients were transduced with high titer lentivirus for HIF1A-sh (mixture of HIF1A-sh1 plus HIF1A-sh2) or scrambled control (Sr-sh) by centrifugation at 2500 rpm for 2 h, and were then cultured for additional 30 h before given to mice. The GFP expression in the sh construct was photographed 24 h after transduction and represents transfection efficiency. B. Silencing efficiency of HIF1A-sh was determined by fluorescence microscopy of HEK293FT cells 24 h after co-transfection with either HIF1A-sh plus HIF1α-P2A-RFP, or Sr-sh plus HIF1α-P2A-RFP as a parallel control. C. Western blots are shown for U251 WT and HIF1A-KO cells incubated in normoxic or severe hypoxic incubator for 8 or 48 h before being lysed for Western blot. D. CoCl2 activated the promoter activities of PDGFRA and PDGFD. HEK293FT cells were transfected with PDGFRA or PDGFD promoter GFP reporter plasmids for 8 h, then treated with CoCl2 for an additional 16 h. GFP expression was then visualized by fluorescence microscopy as shown in the top panels, and quantitated b [file 13046_2021_2082_MOESM1_ESM.docx]

**Supplemental data**

**RNA-Sequencing (RNA-Seq) analysis**

RNA-Seq analysis was performed by Vazyme Biotech Co. Ltd, Nanjing, China. Briefly, three separately isolated RNAs from each of WT U251 line and HIF1A KO U251 lines were processed according to the instructions of RNA library preparation, sequencing and bioinformatics. We used Cuffdiff program to find differentially-expressed genes, which were identified with p value ≤0.05 and a fold-change ≥2 in WT versus KO cells.

**Tissue Fluorescence Staining**

Five micrometer thick conventional sections of formalin-fixed paraffin-embedded glioblastoma tissue arrays (GL803 for PDGFRα and GL805 for PDGF-D, US Biomax) were de-paraffined, rehydrated, and antigen retrieved by boiling in a 0.5% urea Tris-HCl buffer in microwave for 10 min. After permeabilization in 0.3% Triton-X100 PBS solution, the sections were stained using rabbit HIF1α antibody, or together with mouse PDGFRα or goat PDGF-D antibodies overnight at 4℃. Prior to staining the tissue array, strain-matched isotype control antibodies were used to stain trial tissue samples to confirm the primary antibody staining specificity. For co-immunofluorescence staining, secondary donkey anti-rabbit Alexa-Fluor 594 and donkey anti-goat Alexa-Fluor 488 were applied for 2 hours and, after washing, DAPI staining was applied for 5 minutes.

**Western blot**

Cells in culture plates were washed once with PBS and directly lysed with 2xSDS loading buffer containing the reducing reagent dithiothreitol. Lysate was sonicated and centrifuged at 13,000 rpm for 10 min in bench-top centrifuge. Supernatant was heated at 95°C for 3 min before running with 10% SDS-PAGE gel and then transferred to PVDF membrane. The membrane was blocked with 5% dry milk in Tris-HCl buffer for 1 hour at room temperature and then incubated overnight with primary antibody at 4℃ with gently shaking. After blotting with horseradish peroxidase-conjugated secondary antibody for 3 hrs at room temperature, the membrane was washed and visualized with Amersham enhanced ECL detection kit.

**RNA extraction and Reverse Transcription PCR (RT-PCR)**

Freshly isolated RNA with TRIZOL reagent was used for RT-PCR performed using Revert Aid First Strand cDNA Synthesis kit (Thermo Scientific). Briefly, five micrograms of total RNA were used for cDNA synthesis with a random hexane primer and reverse transcriptase, according to the manufacturer’s instructions. Quantitative PCR (qPCR) was performed according to manufacturer's recommendations (Applied Biosystems).

**Cell Invasion Assay**

Boyden Chamber was used to assay invasive migration according to manufacturer’s instructions of cell invasion assay kit (ECM550, Millipore). Briefly, 1x10^5^ cells in 500 µl complete culture medium were added to the top chamber of a trans-well that was inserted into 750 µl complete culture medium in the bottom chamber. After 4 to 5-day culture, the cells in the top chamber of the trans-well were wiped out completely and then the chamber was immediately inserted to staining solution for 5 minutes. After gently completely washing out staining solution in the top chamber of the insert by tap water, photos of the washed insert were taken under microscope.

**MTT assay for cell viability**

The GBM cells (1x10^5^/well) were seeded in a 24-well plate and cultured 24 hours before treatment with different concentrations of Echinomycin for additional 48 hours. Ten microliters of 10 mg/mL 3-(4,5-dimethylthiazol-2-yl)-2,5-diphenyltetrazolium bromide (MTT, Sigma) in PBS was added to each well. Plates were returned to the cell culture incubator for 2 to 4 hours. The resulting MTT formazan crystals were pelleted and dissolved by adding 150 μl DMSO. Absorbance was measured at 490 nm.

**Supplemental Figures**

**Figure S1. Sequencing of PCR products of genomic DNAs of HIF1A knocked out in U251 cells by HIF1A sgRNA**

**Figure S2. HIF1A KO in U87MG cells inhibits cell growth. A, B.** HIF1A KO U87 cells grow slower than WT cells. HIF1A in U87MG cells was knocked out by Cas9-sgRNA method, which was identified by DNA sequencing of PCR product and by Western-blot (A). WT and HIF1A KO U87MG cells were seeded 1x10^4^ /well in a 6-well plate and cultured for 5 days prior to crystal violet staining to observe colony growth (B). **C.** NSG mice implanted with HIF1A KO U87MG cells survive longer than WT U251 cells. NSG mice were intracranially implanted with HIF1A KO or WT U87MG cells (5x 10^4^/mouse) and Kaplan-Meier survival curves were estimated.

A B

**
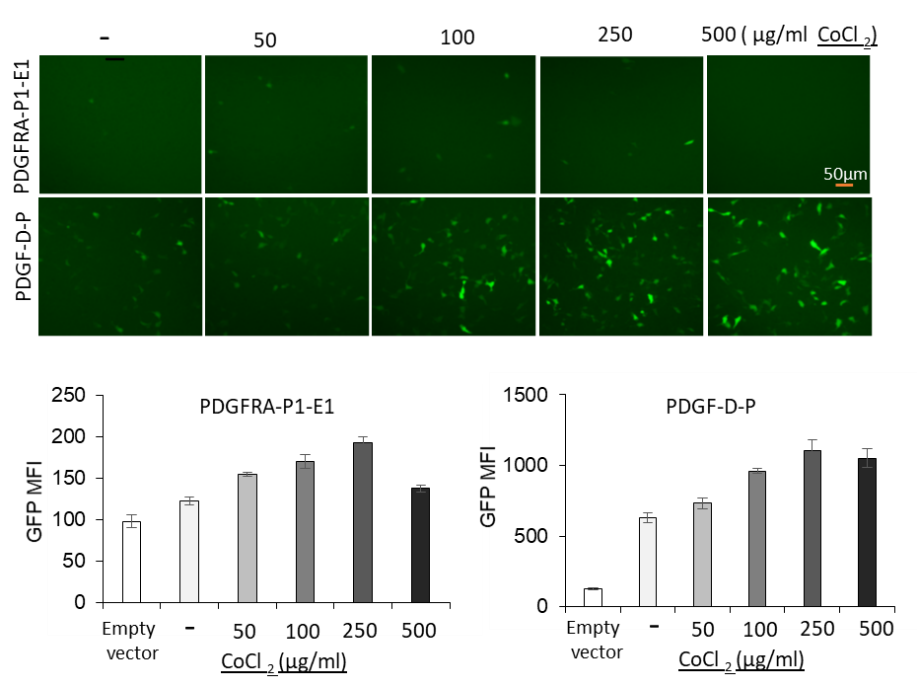

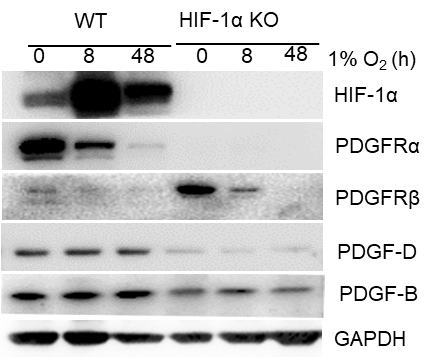
**C D

**Figure S3. HIF1α is required for the expression of PDGFD and PDGFRA under normoxia and mild hypoxia. A.** The efficiency of lentiviral infection of primary Glio-1 and Glio-2. Freshly isolated cells from tumor recipients were transduced with high titer lentivirus for *HIF1A*-sh (mixture of *HIF1A*-sh1 plus *HIF1A*-sh2) or scrambled control (Sr-sh) by centrifugation at 2500 rpm for 2 hours, and were then cultured for additional 30 hours before given to mice. The GFP expression in the sh construct was photographed 24 hours after transduction and represents transfection efficiency. **B.** Silencing efficiency of *HIF1A*-sh was determined by fluorescence microscopy of HEK293FT cells 24 hours after co-transfection with either *HIF1A*-sh plus HIF1α-P2A-RFP, or Sr-sh plus HIF1α-P2A-RFP as a parallel control. **C.** Western blots are shown for U251 WT and *HIF1A*-KO cells incubated in normoxic or severe hypoxic incubator for 8 or 48 hours before being lysed for Western blot. **D.** CoCl_2_ activated the promoter activities of PDGFRA and PDGFD. HEK293FT cells were transfected with PDGFRA or PDGFD promoter GFP reporter plasmids for 8 hours, then treated with CoCl_2_ for an additional 16 hours. GFP expression was then visualized by fluorescence microscopy as shown in the top panels, and quantitated by flow cytometry, summarized below. Bar graphs represent mean fluorescence intensity (MFI) readings ± SD for triplicate wells at each dose.

**Figure S4. HIF1α, PDGFRα and PDGF-D are frequently co-expressed in GBM. A.** Co-expression of HIF1α and PDGFRα in GBM. Microarray of brain tumor with adjacent normal tissues was co-stained with primary rabbit HIF1α antibody and mouse PDGFRα antibody, and with secondary antibodies goat anti-rabbit Alexa-Fluor 594 and goat anti-mouse Alexa-Fluor 488 after washing away primary antibodies. DAPI was used to visualize nuclei (blue). **B.** Summary of microarray cases with double positive staining for HIF1α and PDGFRα, which was presented as percentage of total tumor subtype cases. ANB, adjacent normal brain tissue; OD, oligodendrocytoma; OA, oligoastrocytoma; AA, anaplastic astrocytoma; MB, medulloblastoma; EP, ependymoma. Data shown are representative of two independent experiments. **C.** Correlation of staining intensity for HIF1α with PDGFRα in GBM tissue microarray. IF staining of a 35 cases tissue microarray of GBM and 5 cancer adjacent normal cerebral tissue was performed with PDGFRα and HIF1α antibodies together as described in A. The double positive stains of cases with low, moderate, high, or very high scores were analyzed for the correlation of HIF1α with PDGFRα. **D.** Co-expression of HIF1α and PDGF-D in GBM. Sections of brain tumor with adjacent normal brain tissue microarray were co-stained with primary rabbit HIF1α antibody and goat PDGF-D antibody, and with secondary antibodies donkey anti-rabbit Alexa-Fluor 488 and donkey anti-goat Alexa-Fluor 594 after washing away primary antibodies. DAPI was used to visualize nuclei (blue). The correlation of staining intensity for HIF1α and PDGF-D are shown. **E.** Correlation of staining intensity for HIF1α with PDGFD in GBM tissue microarray. IF staining of a 38-cases tissue microarray of GBM and 6 of normal cerebrum tissues was performed with PDGF-D and HIF1α antibodies as described in D. The double positive stains of 23/38 cases with low, moderate, high, or very high scores were analyzed for the correlation of HIF1α with PDGF-D.

**Figure S5. TP53 mutation did not affect GBM response to Echinomycin**. **A.** PDGF-DD levels in the medium of *HIF1A* KO or WT U251 cells. Levels of released PDGF-D protein were measured by ELISA. **B.** Echinomycin induced apoptosis of U251 cells. Annexin V staining was performed on U251 cells that were treated for 48 hours with different concentrations of Echinomycin. **C, D.** *HIF1A*-KO or *PDGFRA*-KO cells are resistant to Echinomycin. WT, *HIF1A*-KO or *PDGFRA*-KO U251 cells were treated with different concentrations of Echinomycin for 72 hours prior to determining cell viability by MTT assay. **E.** Primary Glio-1 cells have an R273H mutation of TP53 which is also carried by U251 cells. Sequence chromatograms are shown with arrows indicating R273H mutation

**
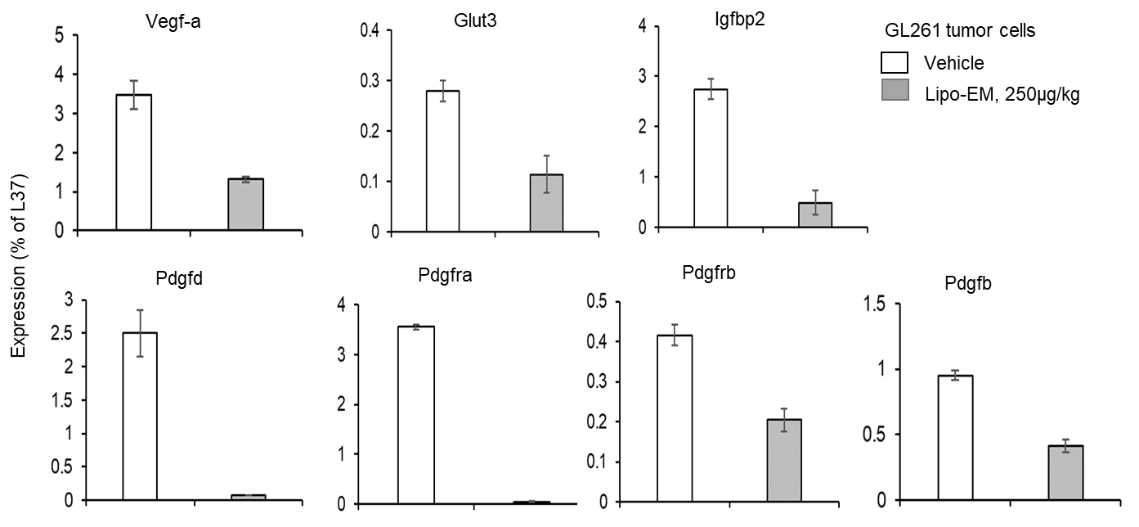
**

**Figure S6. Echinomycin reduced the expression of HIF1α target genes.** Empty vector-transfected GL261 brain tumor cells were orthotopically transplanted to recipient mice, and the mice were treated with vehicle or LEM as detailed in methods. Twenty-four hours after the final dose, the tumor cells were isolated and the cDNA was used to perform qRT-PCR.

**Table S1 Characteristics of Clinical Glioma Samples**

**Table S2. RNA-Seq data of growth factor related genes expressed in WT and HIF1α KO in U251 cells**


**Table S3. RNA-Seq data of metabolism related genes expressed in WT and HIF1α KO in U251 cells**
